# Coupling Electronic and Phonon Thermal Transport in Poly(3,4-ethylenedioxythiophene)-poly(styrenesulfonate) Nanofibers

**DOI:** 10.3390/nano12081282

**Published:** 2022-04-09

**Authors:** Lan Dong, Chengpeng Bao, Shiqian Hu, Yuanyuan Wang, Zihua Wu, Huaqing Xie, Xiangfan Xu

**Affiliations:** 1 School of Energy and Materials, Shanghai Polytechnic University, Shanghai 201209, China; donglan@sspu.edu.cn (L.D.); 20191510071@sspu.edu.cn (C.B.); wangyuanyuan@sspu.edu.cn (Y.W.); hqxie@sspu.edu.cn (H.X.); 2 Shanghai Engineering Research Center of Advanced Thermal Functional Materials, Shanghai Polytechnic University, Shanghai 201209, China; 3 Department of Physics, Yunnan University, Kunming 650091, China; shiqian@ynu.edu.cn; 4 Center for Phononics and Thermal Energy Science, China-EU Joint Center for Nanophononics, School of Physics Science and Engineering, Tongji University, Shanghai 200092, China

**Keywords:** electrical conductivity, electronic contribution, phonon contribution, Lorenz number, PEDOT:PSS nanofiber

## Abstract

The thermal transport of Poly(3,4-ethylenedioxythiophene)-poly(styrenesulfonate) (PEDOT:PSS) nanofiber is contributed by the electronic component of thermal conduction and the phonon component of thermal conduction. The relationship between the electrical conductivity and thermal conductivity of these conducting polymers is of great interest in thermoelectric energy conversation. In this work, we characterized the axial electrical conductivities and thermal conductivities of the single PEDOT:PSS nanofibers and found that the Lorenz number *L* is larger than Sommerfeld value *L*_0_ at 300 K. In addition, we found that the *L* increased significantly in the low-temperature region. We consider that this trend is due to the bipolar contribution of conducting polymers with low-level electrical conductivity and the increasing trend of the electronic contribution to thermal conductivity in low-temperature regions.

## 1. Introduction

Conducting polymers belong to conjugated polymers that contain unsaturated carbon backbones and are expressed as delocalization of π-electrons along the long molecular chains [1]. The overlapping π-electrons orbitals between intrachains and interchains provide channels for carrier transport and energy exchange [2]. The carrier transport of conjugated polymer could be affected by the doping process [3,4,5]. In contrast with inorganic semiconductors, the doping of conducting polymers usually introduces large molecular dopants that could change the ordered chain stacking [6]. It is well known that conducting polymers have excellent stability and environmental friendliness that attract extensive research interests in various application fields including photovoltaic technology [7], thin film transistor [8,9], memory storage [10], thermoelectric cooling, and power generation device [11,12,13], etc. Conducting polymers are generally considered as the potentially thermoelectric system due to its intrinsic ultra-low thermal conductivity, mechanical flexibility, and low-cast fabrication [14]. Poly(3,4-ethylenedioxythiophene)-poly(styrenesulfonate) (PEDOT:PSS) is one of the most common thermoelectric polymers which can be doped to realize a wide range electrical conductivities [15]. The regulation of carrier transport of conducting polymers could affect both electrical and thermal transport [16,17,18].

Thermoelectric materials are designed to convert heat energy into electrical energy directly. The dimensionless thermoelectric figure of merit (*ZT)* represents the conversion efficiency of thermoelectric devices, which is usually expressed as *ZT = S*^2^*σT/κ*, where *S*, *σ* and *T* represent the Seebeck coefficient, the electrical conductivity, and the ambient temperature, respectively. The *κ* is the total thermal conductivity [19]. The total thermal conductivity (*κ*) can be expressed as *κ* = *κ_ph_* + *LσT* according to the Wiedemann–Franz law, the *LσT* represents the electronic contribution of *κ* and the *κ_ph_* is the phonon contribution to *κ* [20]. The doping process and modulation of molecular structure could influence carrier density and carrier mobility that led to a change of electrical conductivity. N-type polymer FBDPPV thin film with the better ordered molecular packing structure was obtained by adding a small fraction of dopants, via which the electrical conductivity and power factor (*S*^2^*σ*) could be enhanced at the same time [21]. PEDOT:PSS and tellurium-PEDOT:PSS (Te-PEDOT:PSS) thin films present enhanced power factor due to the apparent increase of electric conductivity after H_2_SO_4_ treatment [22]. Both above results focused on optimizing electrical and Seebeck coefficient and ignored the influence on thermal conductivity. Pipe et al. reported the EG-mixed and DMSO-mixed PEDOT:PSS thin films (thickness < 100 nm) and achieved a *ZT* of 0.42 by removing PSS in ethylene glycol (EG) solution [23]. Zapata-Arteaga et al. showed that the molecular doping of the neat PBTTT film increases the electrical conductivity while reducing the thermal conductivity without compromising the crystalline quality, which is similar to the alloy scattering effect in several inorganic systems [24]. Yang et al. reported the decoupled electronic and phonon transport in the single core/shell nanowire of Te-PEDOT:PSS. They found that the origin of the decoupling of charge and heat transport lies in the fact that electrical transport occurs through the organic shell, while the inorganic core drives thermal transport. The highest figure of merit, ZT of Te-PEDOT:PSS nanowire approaches to 0.54 at 400 K [19]. Based on these previous results, it is crucial to clarify the relationship between electrical transport and thermal transport in nanoscale conducting polymers.

Generally, for the bulk conductors and heavily doped semiconductors, the electronic contribution to the thermal conductivity can be well described by the Wiedemann–Franz law (WF law) as *κ*_e_ = *LσT*, the Lorenz number *L* is found to have a small deviation from the Sommerfeld value *L*_0_ = 2.44 × 10^−8^ WΩK^−2^ [25]. While for the nanoscale metal system and lightly doped semiconductor materials, the difference between the Lorenz number *L* and *L*_0_ comes from the phonon contribution to thermal conductivity and the electron-phonon coupling [25,26,27]. Previous research has discussed the relationship between the Sommerfeld value and the Lorenz number of conducting polymers. Liu et al. present the in-plane thermal conductivity of drop-cast DMSO-mixed PEDOT:PSS film as a function of in-plane electrical conductivity. The thickness of this drop-cast film is larger than 20 μm. The result reveals that the electron component of thermal conductivity of PEDOT:PSS film conforms to the WF law with *L* approaches to *L*_0_ [16]. Weathers et al. reported a larger Lorenz number than the *L*_0_ in conducting PEDOT:PSS and PEDOT:Tos thin film, which can be explained by the phonon-assisted hopping mechanism and a bipolar contribution to thermal conductivity [20]. In addition, some previous works had explored the thermal transport properties in polymer nanostructures. Shen et al. suggested the thermal conductivity of the individual polyethylene nanofiber could reach 104 Wm^−1^ K^−1^ with the degree of crystallinity up to 80–90% [28]. While the single Nylon-11 nanofiber with the degree of crystallinity approaches to 36% exhibits the highest thermal conductivity up to 1.6 Wm^−1^ K^−1^ [29]. Singh et al. found that the thermal conductivity of the amorphous polythiophene nanofibers increases as the diameter of the nanofiber decreases [30]. The increasing thermal conductivity is due to the decreasing phonon scattering, caused by the chain alignment in the nanofibers with diameters less than 100 nm. In contrast to previous research, there has not found an apparent relationship between the *σ* and *κ* of the same transport direction (in-plane or cross-plane). Thus, the electronic contribution to thermal conductivity of the nanoscale conducting polymers needs to be further discussed.

In this work, we fabricated the single suspended PEDOT:PSS nanofibers by electrospinning technology and carried out the measurement of axial electrical conductivity and axial thermal conductivity on the same nanofiber. The characterization of electrical and thermal transport in the same direction provides the possibility to testify the validity of the Wiedemann–Franz law in nanoscale conducting polymers. the axial electrical and thermal conductivity in the temperature range from 20 K to 300 K were characterized intThe PEDOT:PSS nanofibers with diameters larger than 100 nm. Thus, the relationship between the Lorenz number *L* and Sommerfeld value *L*_0_ can be revealed in the whole measured temperature range.

## 2. Sample Preparation

**Figure 1:**
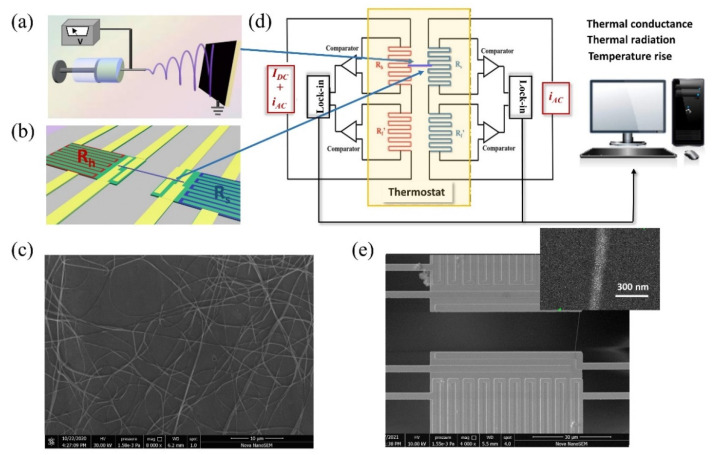
(**a**) the schematic diagram of the electrospinning setup; (**b**) a single nanofiber suspended on the Micro-Electro-Mechanical System (MEMS) device, the nanofiber bridge between the two islands at the center of MEMS device. The heater and thermometer are marked with *R_h_* and *R_s_*; (**c**) a large number of electrospun PEDOT:PSS nanofibers, deposited on the tinfoil substrate. The diameters of the PEDOT:PSS nanofibers are uniform when the spinning voltage is fixed; (**d**) the schematic diagram of measuring axial thermal conductivity of PEDOT:PSS nanofiber by thermal bridge method combined with differential comparison circuit, this method is sensitive enough to characterize both thermal conductance and thermal radiation; (**e**) SEM image of a single PEDOT:PSS nanofiber suspended on the MEMS device, the scale bar is 30 μm. The enlarged SEM image was used to measure the diameter of the sample, and the uncertainty of diameter has been considered in the subsequent measurements. The scale bar of the enlarged image is 300 nm. 1.58e-3 means 1.58 × 10^−3^.

Poly(3,4-ethylenedioxythiophene)-poly(styrenesulfonate) (PEDOT:PSS) aqueous solution, Polyethylene oxide powder (PEO, M_w_ = 900,000) and *N*,*N*-Dimethylformamide solution (DMF) were all purchased from Aladdin Biochemical Technology Co., Ltd. (Shanghai, China). The PEDOT:PSS aqueous solution contains 1.5 wt% PEDOT:PSS in water. PEO powder and DMF were added into PEDOT:PSS aqueous solution to obtain the precursor solution for the electrospinning process. Figure 1a shows the schematic diagram of the electrospinning process. The PEO powder was used to increase the precursor solution’s viscosity, making electrospinning easier to produce nanofiber. During the electrospinning experiments, the spinning distance was fixed to 18 cm and infusion speed to 0.1 mL per minute. In order to modulate the diameters of PEDOT:PSS nanofibers, we changed the viscosity of the precursor solution and alter the spinning voltage from 9 kV to 13 kV. Before the electrical and thermal transport measurement, the PEDOT:PSS nanofibers were prepared on a well-grounded tinfoil substrate which was adhered to the spinning receiver to characterize the morphology and quality of nanofibers. Figure 1c presents a large number of PEDOT:PSS nanofibers fabricated by electrospinning, which is deposited on the tinfoil substrate. We found that the PEDOT:PSS nanofibers both have uniform diameters and small surface roughness.

In order to realize the synchronous measurement for electrical and thermal conductivity, a single PEDOT:PSS nanofiber is electrospun in-situ on a suspended Micro-Electro-Mechanical System (MEMS) device (shown in Figure 1b). The electrodes of the MEMS device were fabricated by platinum thermal evaporation, which were deposited on the silicon nitride (SiN*_x_*) supported beams [31]. The four middle Pt/SiN*_x_* electrodes are usually used to measure the electrical transport of nanofiber through the four-probe method. The twisted Pt/SiN*_x_* electrodes on the two islands are marked with red and blue solid lines in Figure 1b, which are regarded as heater and thermometer for thermal transport measurements. The single PEDOT:PSS nanofiber is required to bridge between the two islands at the center of MEMS device and the axial thermal conductivity of the nanofiber could be characterized by the thermal bridge method [32,33], combining with differential comparison circuit (shown in Figure 1d). One single PEDOT:PSS nanofiber should satisfy the requirements of electrical and thermal measurements at the same time. Figure 1e exhibits the scanning electron microscope (SEM) image of a suspended MEMS device with a single PEDOT:PSS nanofiber after electrical and thermal measurements.

## 3. Measurement

### 3.1. Electrical Conductivity

The axial electrical resistance of the single PEDOT:PSS nanofiber was characterized by the classical four-probe method using four middle electrodes of the MEMS device. The two electrodes near the center of the MEMS device are used to measure the voltage of the sample, and the other two electrodes are used to add the low frequency AC current with amplitude of around 1 μA. The whole MEMS device were put into a high vacuum chamber. The electrical transport and thermal transport measurements can be carried out synchronously in this vacuum chamber. The electrical resistance of the single PEDOT:PSS nanofiber can be characterized under the whole temperature range. The resistances of the nanofiber were detected at least five times to ensure the reliability of the results. The length and diameter of the PEDOT:PSS nanofiber could be obtained from the SEM image. Based on this, the electrical conductivity of the nanofiber could be calculated. The error bar of the electrical conductivity should consider the uncertainty of the nanofiber length, nanofiber diameter, the multiple measurements, and the systemic error from the detection equipment.

### 3.2. Thermal Conductivity

The axial thermal conductivity of a suspended single PEDOT:PSS nanofiber would be measured by thermal bridge method with combination of with differential comparison circuit [34,35]. The design of differential comparison circuit aims to optimize the accuracy of thermal conduction measurement, especially for those low dimensional materials whose thermal conduction is lower than 1 × 10^−10^ W/K. The two adjacent MEMS devices with the same gap and similar islands’ resistances are chosen to build the differential comparison structure. One of the MEMS devices contains the PEDOT:PSS nanofiber and the other is a blank device, which was used as a reference resistor. In thermal bridge measurements, thermal convection and temperature drift could affect the accuracy of thermometer detection. The two MEMS devices were placed into a cryostat with a high vacuum on the order of 1 × 10^−4^ Pa to increase the accuracy. We waited for at least 2 h at each temperature point to ensure the stability of the ambient temperature before thermal transport measurements. The heating DC current were provided by Keithley 6221 to produce the joule heat (*Q_tot_*) and the max heating current approach to 70 μA. The heating DC current and an AC current around 1 μA are both applied to the heater, and the same AC current (~1 μA provided by Keithley 6221) is applied to the thermometer. Both AC currents are used to observe the change of resistance of the heater and thermometer. Here, we mark the heater and thermometer as *R_h_* and *R_s_*. With the gradual increase of DC current, the resistances of *R_h_* and *R_s_* increase accordingly. The change of resistances could reflect the change of temperature ($\Delta T_{h}$ and $\Delta T_{s}$) at the both ends of the PEDOT:PSS nanofiber [36,37]. From these, the thermal conductance of the PEDOT:PSS nanofiber ($G_{\mathrm{nf}}$) could be calculated by $\;G_{b}=Q_{\mathrm{tot}}/\left(\Delta T_{h}+\Delta T_{s}\right)$ and $G_{\mathrm{nf}}=G_{b}\Delta T_{S}/\left(\Delta T_{h}-\Delta T_{s}\right)$, where $G_{b}$ is the thermal conductance of the Pt/SiN*_x_* supporting beams. The thermal conductivity of a single PEDOT:PSS nanofiber (*κ*) could be obtained from $\kappa =G_{\mathrm{nf}}L/A$, where *L* and *A* represent the length and cross-section area of the nanofiber. We consider the cross-section of electrospinning nanofibers to be circular.

## 4. Results and Discussion

**Figure 2:**
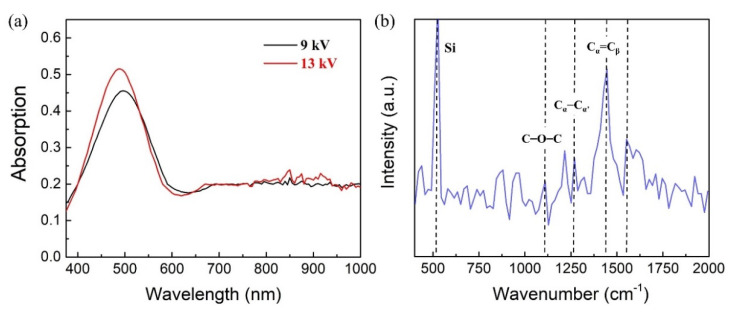
(**a**) the UV-Vis spectra of PEDOT:PSS electrospinning nanofibers deposited on the silicon wafer with spinning voltage at 9 kV and 13 kV; (**b**) the Raman spectrum of the PEDOT:PSS nanofibers deposited on the silicon wafer with 13 kV spinning voltage, the 520 cm^−1^ peak is the characterize peak of the silicon.

Figure 2a presents UV-Vis spectra of PEDOT:PSS electrospinning nanofibers deposited on the silicon wafer. The UV-Vis spectrum of PEDOT:PSS nanofibers with different spinning voltages (9 kV and 13 kV) shows a similar trend with relatively strong absorptions in the UV at 400–700 nm. The higher energy transition can be assigned to π–π* transitions in the PEDOT:PSS backbone [38,39]. It shows that the PEDOT:PSS nanofibers with different spinning voltages have the same light absorption capacity in the whole measured wavelength range, and thus the change of spinning voltage will not affect the molecular structure of the nanofiber. Figure 2b presents the Raman spectrum of the PEDOT:PSS nanofibers deposited on the silicon wafer with the 13 kV spinning voltage. The 520 cm^−1^ characteristic peak represents silicon. For PEDOT:PSS, the prominent peak with wavenumber at 1445 cm^−1^ represents the symmetric C_α_=C_β_ stretching vibrations. The characteristic peaks with wavenumbers at 1269 cm^−1^,1109 cm^−1^, and 1578 cm^−1^ assign to the C_α_–C_α’_ inter-ring stretching vibrations, the C–O–C deformation, and the quinoid structure. The 1445 cm^−1^ peak is the most significant peak of the PEDOT:PSS that is usually used to reflect the level of oxidation of the PEDOT:PSS system [40].

**Figure 3:**
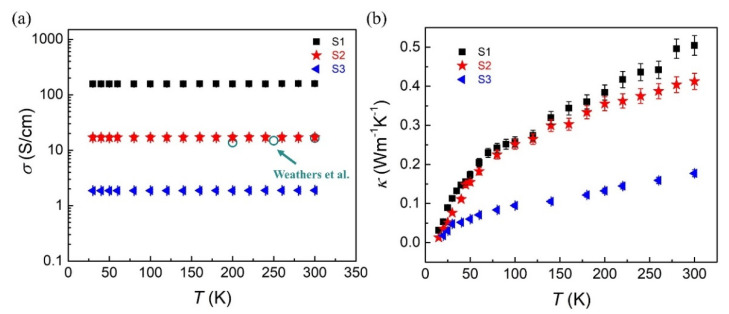
(**a**) the axial electrical conductivities of S1, S2, and S3 nanofibers, which exhibit a slight enhance as the temperature increase from 20 K to 300 K. The length of the S1, S2, and S3 are 20.3 μm, 15.7 μm, and 15.3 μm and the diameter of the S1, S2 and S3 are 160 nm, 171 nm, and 138 nm, respectively. The dark green circles represent the increased trend of electrical conductivity of PEDOT:PSS fiber in previous result [20]; (**b**) the axial thermal conductivities of three samples increase as the temperature increase. The increasing trend of thermal conductivity proved that PEDOT:PSS nanofibers are amorphous dominated molecular structures.

To introduce the axial electrical and thermal transport of single PEDOT:PSS nanofiber, we prepared the electrospinning precursor solution with different PEO proportions. PEO is used to adjust the viscosity of the PEDOT:PSS aqueous solution and realize the different electrical conductivity of nanofibers within a certain range. As shown in Figure 3a, the axial electrical conductivities of three PEDOT:PSS nanofibers can be characterized in the temperature range from 20 K to 300 K. We labeled the three PEDOT:PSS nanofibers S1, S2, and S3, respectively. The length of the S1, S2, and S3 are 20.3 μm, 15.7 μm, and 15.3 μm and the diameter of the S1, S2, and S3 are 160 nm, 171 nm, and 138 nm, respectively. The electrical conductivities of the PEDOT:PSS nanofibers exhibit a slight increase as the temperature increase, which shows the electrical transport properties similar to semiconductors. The electrical conductivities of the three samples at 300 K reach 158.8 S/cm, 17.1 S/cm, and 1.89 S/cm, respectively. From there, we compared the increased conductivity trend of the S2 with previous results [20] and found both results are in good agreement with each other (shows in Figure 3a). It can be proved that the electrical conductivities of the electrospinning PEDOT:PSS nanofibers measured by the four-probe method in our experiments are completely reliable.

Figure 3b exhibits the axial thermal conductivities of PEDOT:PSS nanofibers with the same temperature range as electrical transport measurements. The increasing thermal conductivity of the single PEDOT:PSS nanofiber could be found during the whole temperature region. It can be proved that there has no obvious phonon-phonon Umklapp scattering in the PEDOT:PSS nanofiber. This increasing trend of thermal conductivity indicates the amorphous dominated molecular structures in PEDOT:PSS nano-system. Dominated energy transport of the PEDOT:PSS nanofibers focuses on the inter-chain and the intra-chain phonon hopping transport [41]. Considering that the thermal conduction of the single nanofiber (~10^−10^ W/K) is less than one order of magnitude higher than the thermal radiation between the heater and thermometer of MEMS device, we should characterize the thermal radiation of the blank MEMS device and subtract the thermal radiation from the total thermal conduction to obtain the actual thermal conduction of the single PEDOT:PSS nanofiber. The thermal conductivity of the S1, S2, and S3 nanofiber at 300 K is 0.46 Wm^−1^ K^−1^, 0.35 Wm^−1^ K^−1^, 0.24 Wm^−1^ K^−1^, respectively.

According to the previous work, for the polymer nanofibers’ diameters smaller than 100 nm, the thermal conductivity of insulating polymer nanofiber would increase as the diameter of nanofiber decrease, which is due to the chain alignment of the nanofibers could decrease the phonon scattering [34]. The chain alignment usually occurs in polymer nanofibers where the diameter is less than 100 nm. While for the diameter of the polymer nanofiber larger than 100 nm, the chain alignment will disappear and the thermal transport properties of this larger diameter nanofiber will much similar to that of the bulk polymer (eg. thermal conductivity of bulk PEDOT is around 0.1 Wm^−1^ K^−1^ at room temperature). In this experiments, we found that the axial thermal conductivities of three PEDOT:PSS nanofibers are both larger than the thermal conductivity of bulk polymers. According to the previous conclusion, there has no chain alignment in the three PEDOT:PSS nanofibers due to the diameters larger than 100 nm. This enhanced thermal conductivities of the conducting PEDOT:PSS nanofibers are most likely caused by the electronic contribution of total thermal conductivity. As the experiments could characterize the electrical conductivity and thermal conductivity along the same transport direction (axial direction) of the same suspended PEDOT:PSS nanofiber, there has an opportunity to verify the validity of the Wiedemann–Franz law in PEDOT:PSS nano-structure.

**Figure 4:**
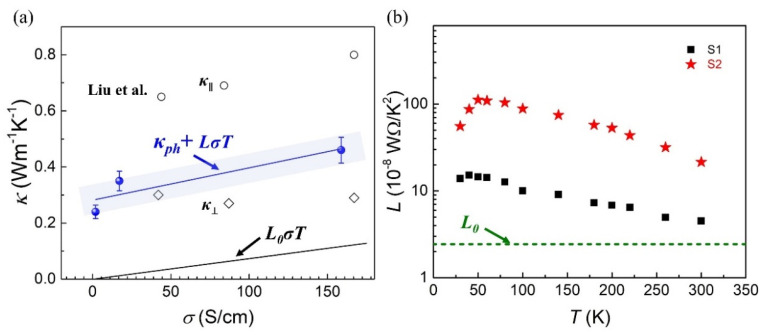
(**a**) the axial thermal conductivities of the S1, S2, and S3 nanofibers increase as the electrical conductivity changes by two orders of magnitude at 300 K. Blank circles and diamonds represent the in-plane thermal conductivity (κ_∥_) and the cross-plane thermal conductivity (κ_⊥_) of the PEDOT:PSS film [16]; (**b**) the calculated Lorentz number *L* of S1 and S2 in the whole measured temperature range. The olive-green dotted line represents the Sommerfeld value *L*_0_.

Figure 4a shows the thermal conductivities at 300 K of the three PEDOT:PSS nanofibers (blue balls) which increase as the electrical conductivities change from 1.89 S/cm to 158.8 S/cm. For the conducting polymers, both electrons and phonons can carrier heat to realize energy transport. The thermal conductivity of conducting polymer is contributed by *κ_e_* and *κ_ph_*. In our experiments, the S3 nanofiber has an ultra-low electrical conductivity (1.89 S/cm) at 300 K, resulting in an almost negligible electronic contribution to *κ* of PEDOT:PSS nanofiber. Thus, we could consider the thermal conductivity of S3 (0.24 Wm^−1^ K^−1^ at 300 K) as the phonon contribution to *κ* (*κ_ph_*). Due to the utterly consistent sample preparation process, *κ_ph_* was considered as the same in each PEDOT:PSS nanofiber. The relationship between the estimated electronic contribution to the total thermal conductivity of PEDOT:PSS nanofiber (*κ*) can be expressed as *κ* = *κ_ph_* + *LσT* according to the Wiedemann–Franz law. The blue line in Figure 4a is used to fit the thermal conductivities of three PEDOT:PSS nanofibers whose electrical conductivities vary by two orders of magnitude. The Lorentz number *L* will equal to the Sommerfeld value (*L*_0_) as the Wiedemann–Franz law is valid, where the thermal conductivity of free carriers could be expressed as *κ_e_* = *L*_0_*σT*. The *L*_0_*σT* is shown in Figure 4a used the black line. Our experiments found that the *L* is larger than *L*_0_, due to the contribution of electronic component of the thermal conductivity. A similar result was found in Liu’s work, where the in-plane thermal conductivities of the PEDOT:PSS films increase as the electrical conductivities increase. It realizes the uniformity of the WF law (shown in Figure 4a as empty circles) [16]. In addition to the in-plane thermal conductivities of the PEDOT:PSS films, the cross-plane thermal conductivities were also measured simultaneously in Liu’s work (shown in Figure 4a as empty diamonds), and the cross-plane thermal transport exhibited an obvious electrical conductivity independence [16]. The difference in phonon contribution to κ between PEDOT:PSS film and our nanofiber might be due to the different pure PEDOT:PSS solution and the different method for thermal transport measurements.

As shown in Figure 3, the electrical conductivity of the S3 nanofiber changes slightly during the whole measurement temperature range. Considering the ultra-low electrical conductivity of S3 is around 1.8 S/cm in the whole temperature range, we believe that the total thermal conductivity of S3 is almost entirely contributed to the lattice component of the thermal conductivity. To estimate the electronic component of the thermal conductivity in S1 and S2 nanofibers, we calculated the Lorentz number *L* in the temperature range of 20 K to 300 K (shows in Figure 4b). The Lorentz number of S1 exhibit a significant decrease compared with that of S2. It is proved that the higher electrical conductivity would increase the proportion of the electronic contribution to *κ* in PEDOT:PSS nano-structure. The Lorentz number of S1 is closer to *L*_0_, which indicates that the conducting polymer with higher electrical conductivity is more likely to accord with the Wiedemann–Franz law. As shown in Figure 4b, the *L* of PEDOT:PSS nanofibers deviated more from *L_0_* in the low temperature range. There are two possible mechanisms could lead to the higher *L* than Sommerfeld value (*L*_0_): (1) the higher Lorentz number *L* can be caused by a more considerable bipolar contribution of conducting polymers with low level electrical conductivity. For the semiconductor system, the low electrical conductivity is due to the position of Fermi energy placed close to the mid-gap so as to realize the bipolar contribution in the thermoelectric materials. Previous work has demonstrated that the additional bipolar contribution could increase the Lorentz number *L* of suspended Bismuth Telluride nanoplates [42]. Similar situation can be compared with that in conducting polymers; (2) we could find in Figure 3a,b as the temperature decrease from 300 K to 20 K, the electrical conductivities of S1 and S2 decrease by 1.36% and 0.59%, respectively. Meanwhile, the thermal conductivities of S1 and S2 decrease by 76% and 77%, respectively. This significant decrease in thermal conductivity in the low temperature region (and the electrical conductivity is almost constant) will lead to the increasing trend of the electronic contribution to *κ* and directly guide the higher Lorentz number *L* in the low temperature region. It can be found in Figure 4b, the Lorentz number *L* decreases close to *L_0_* as the temperature increases to 300 K, which might be explained by the unique hopping transport mechanism of the polymer system [20]. The intra-chain and inter-chain hopping channels will assist the realization of electronic contribution to thermal transport in the low temperature range.

## 5. Conclusions

The single suspended PEDOT:PSS nanofiber was prepared on the MEMS device by the electrospinning process. We measured the axial electrical conductivities and thermal conductivities of the three PEDOT:PSS nanofibers (S1, S2, and S3) in the whole temperature range. From this, we found that the electrical conductivities of the nanofibers exhibit a slight increase during the whole temperature region. In contrast, the thermal conductivities increase significantly as the temperature change from 20 K to 300 K. In addition, the thermal conductivities of the three samples at 300 K exhibit a monotonic increase with electrical conductivities increase from 1.89 S/cm to 158.8 S/cm and the calculated Lorentz number *L* according to the Wiedemann–Franz law is observed to be larger than the Sommerfeld value *L*_0_. For the S1 and S2, we found that the *L* shows decreased trend as the temperature increase. We believe this trend is due to the bipolar contribution of conducting polymers with low level electrical conductivity and the increasing trend of the electronic contribution to *κ* in low temperature region.

## Data Availability

Not applicable.
